# Supplementary material for: Inhibition of UBA6 by inosine augments tumour immunogenicity and responses
Source: Nat Commun. 2022 Sep 15;13:5413. doi: 10.1038/s41467-022-33116-z (PMC9478149; doi:10.1038/s41467-022-33116-z)
Supplement: Supplementary file 1 — Supplementary Information [file 41467_2022_33116_MOESM1_ESM.pdf]

Supplementary Materials for

Inhibiting tumour cell UBA6 expression by inosine augments tumour immunogenicity and response to immune checkpoint inhibition

Lei Zhang,[1,2,†] Li Jiang,[3,†] Liang Yu,[4,†] Qin Li,[5,†] Xiangjun Tian,[6] Jingquan He,[7] Ling Zeng,[1,2] Yuqin Yang,[8] Chaoran Wang,[5] Yuhan Wei,[5] Xiaoyue Jiang,[5] Jing Li,[9] Xiaolu Ge,[1,2] Qisheng Gu,[4] Jikun Li,[4] Di Wu,[10,11] Anthony J Sadler,[12,13] Di Yu,[14] Dakang Xu,[15] Yue Gao,[16]* Xiangliang Yuan,[15]* and Baokun He[1,2,17]*

Correspondence to: baokun.he@shgh.cn (B.H.); yuanxiangliang@gmail.com (X.Y.); gaoyue@bmi.ac.cn (Y.G.)

This file includes:

Supplementary Figure 1 to 8

Supplementary Table 1

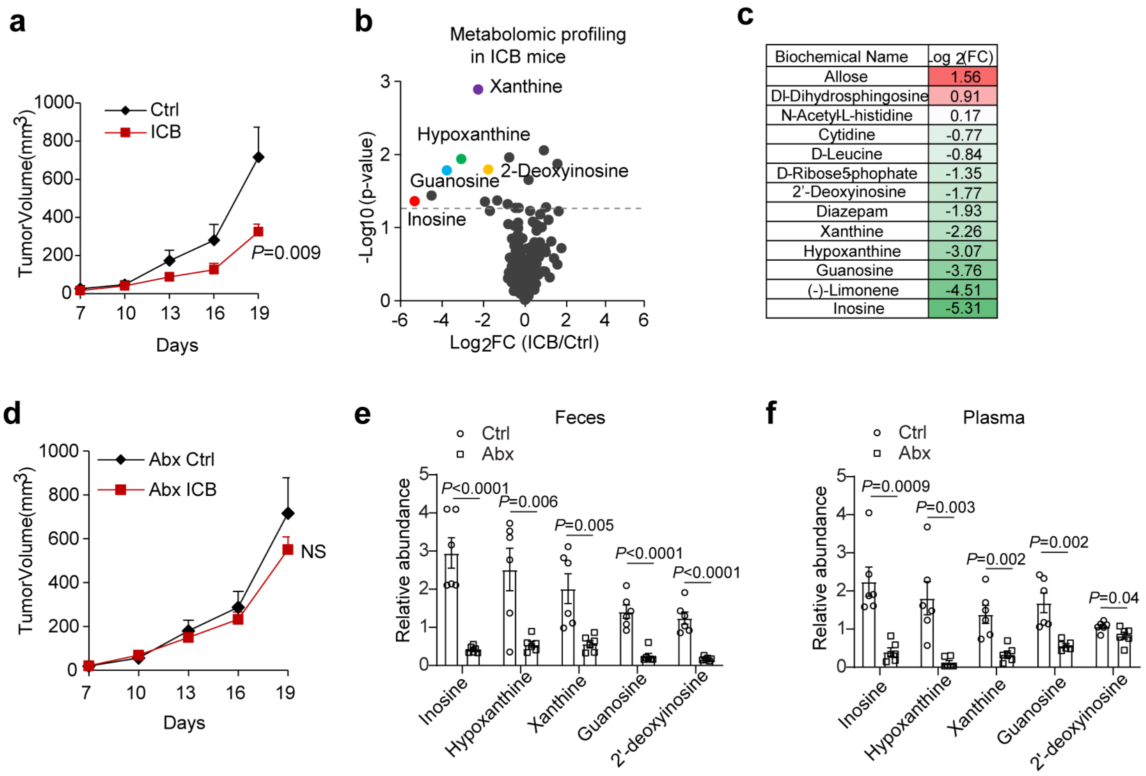

Supplementary Figure 1. Purine metabolism is changed by ICB treatment in mice. (**a**) B16-F0 tumour volume in SPF mice with IgG2a (Ctrl) (n=6) or anti-CTLA4+anti-PD1 antibody (ICB) (n=10) treatment after tumour implantation. (**b**) Volcano plot of plasma metabolomics for B16-F0 tumour-bearing mice (ICB/Ctrl) (n=7). (**c**) Heat map of 13 plasma metabolites ($P< 0.05$) in B16-F0 tumour-bearing SPF mice (ICB/Ctrl) (n=7). (**d**) B16F0 tumour volume in Abx-treated SPF mice with IgG2a (Abx Ctrl) (n=8) or anti-CTLA4+anti-PD1 antibody (Abx ICB) (n=13) treatment after tumour implantation. (**e-f**) The relative abundance of purine metabolites in feces (e) and plasma (f) of SPF mice (Ctrl) and antibiotic-treated mice (Abx) (n=6).

Data are presented as Mean ± s.e.m. Statistical significance was determined by Two-sided Student's t-test (a, d) or one-way ANOVA and Tukey test for multiple comparisons (e, f). The *P*-values in b were based on log-rank tests. The distribution *P*-value is indicated in the figures. Source data are provided as a Source Data file.

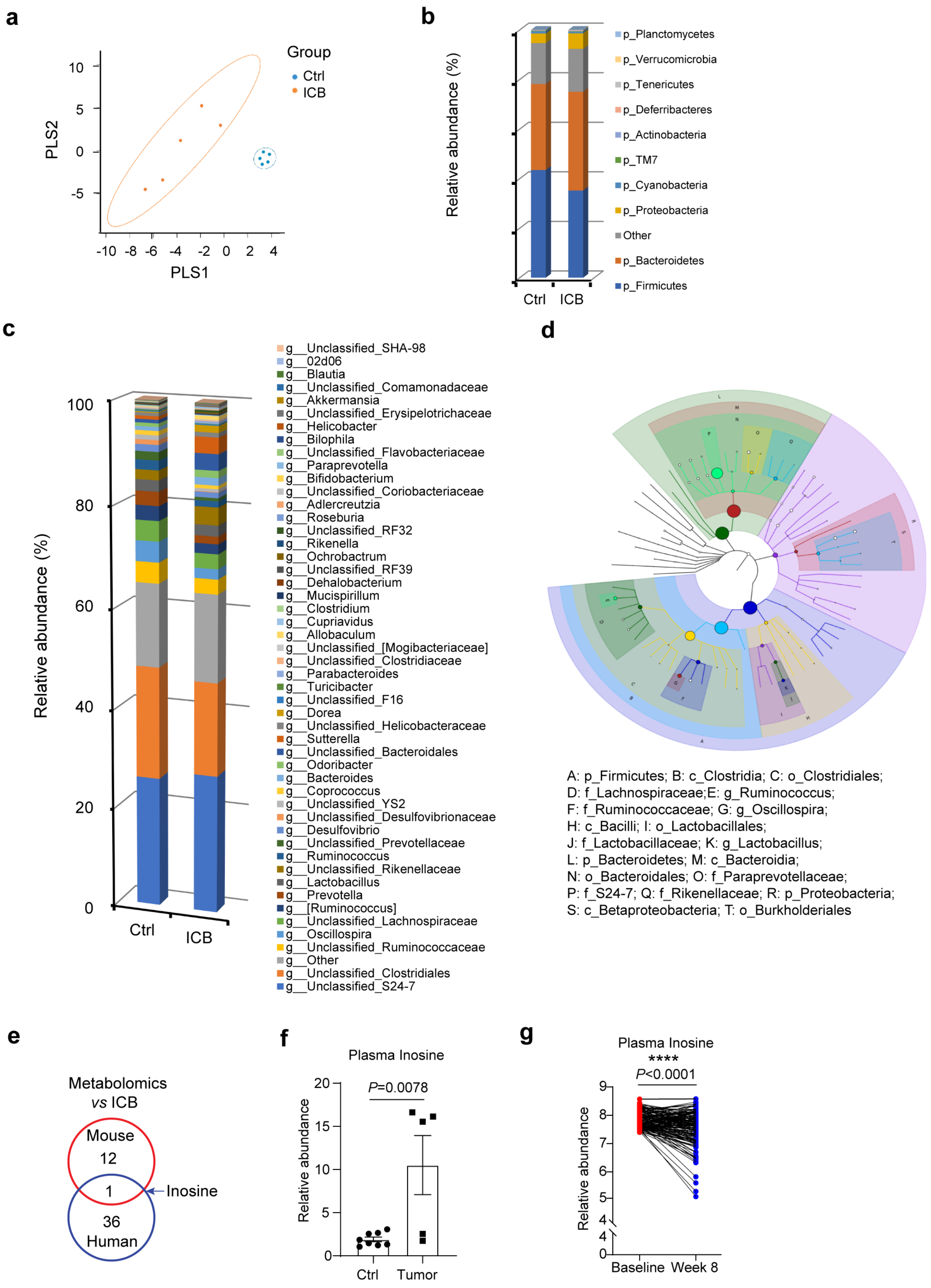
a
Group
Ctrl
ICB
PLS2
PLS1
10
5
0
-5
-10 -8 -6 -4 -2 0 2 4
b
Relative abundance (%)
Ctrl ICB
p_Planctomycetes
p_Verrucomicrobia
p_Tenericutes
p_Deferribacteres
p_Actinobacteria
p_TM7
p_Cyanobacteria
p_Proteobacteria
Other
p_Bacteroidetes
p_Firmicutes
c
Relative abundance (%)
100
80
60
40
20
0
Ctrl
ICB
g__Unclassified_SHA-98
g__02d06
g__Blautia
g__Unclassified_Comamonadaceae
g__Akkermansia
g__Unclassified_Erysipelotrichaceae
g__Helicobacter
g__Bilophila
g__Unclassified_Flavobacteriaceae
g__Paraprevotella
g__Bifidobacterium
g__Unclassified_Coriobacteriaceae
g__Adlercreutzia
g__Roseburia
g__Unclassified_RF32
g__Rikenella
g__Ochrobactrum
g__Unclassified_RF39
g__Dehalobacterium
g__Mucispirillum
g__Clostridium
g__Cupriavidus
g__Allobaculum
g__Unclassified_[Mogibacteriaceae]
g__Unclassified_Clostridiaceae
g__Parabacteroides
g__Turicibacter
g__Unclassified_F16
g__Dorea
g__Unclassified_Helicobacteraceae
g__Sutterella
g__Unclassified_Bacteroidales
g__Odoribacter
g__Bacteroides
g__Coprococcus
g__Unclassified_YS2
g__Unclassified_Desulfovibrionaceae
g__Desulfovibrio
g__Unclassified_Prevotellaceae
g__Ruminococcus
g__Unclassified_Rikenellaceae
g__Lactobacillus
g__Prevotella
g__[Ruminococcus]
g__Unclassified_Lachnospiraceae
g__Oscillospira
g__Unclassified_Ruminococcaceae
g__Other
g__Unclassified_Clostridiales
g__Unclassified_S24-7
d
A: p_Firmicutes; B: c_Clostridia; C: o_Clostridiales;
D: f_Lachnospiraceae;E: g_Ruminococcus;
F: f_Ruminococcaceae; G: g_Oscillospira;
H: c_Bacilli; I: o_Lactobacillales;
J: f_Lactobacillaceae; K: g_Lactobacillus;
L: p_Bacteroidetes; M: c_Bacteroidia;
N: o_Bacteroidales; O: f_Paraprevotellaceae;
P: f_S24-7; Q: f_Rikenellaceae; R: p_Proteobacteria;
S: c_Betaproteobacteria; T: o_Burkholderiales
e
Metabolomics
vs ICB
Mouse
12
1
36
Human
Inosine
f
Plasma Inosine
P=0.0078
Relative abundance
20
15
10
5
0
Ctrl
Tumor
g
Plasma Inosine
****
P<0.0001
Relative abundance
9
8
7
6
5
4
4
0
Baseline
Week 8

Supplementary Figure 2. Gut microbiota dysbiosis and plasma inosine levels are associated with ICB immunotherapy. (**a**) Partial least squares discriminant analysis (PLS-DA) score scatter plot of the overall bacterial community in B16F0 tumour-bearing SPF mice with Ctrl or ICB treatment (n=5). (**b**) The relative abundance of total bacteria at the phylum level in B16-F0 tumour-bearing SPF mice with Ctrl or ICB treatment (n=5). (**c**) The relative abundance of predominant bacteria (top 50) at the genus level in B16F0 tumour-bearing SPF mice with Ctrl or ICB treatment (n=5). (**d**) Cladogram of the gut microbiota from B16F0 tumour-bearing SPF mice with Ctrl or ICB treatment (n=5). (**e**) Venn diagrams showing that inosine is the overlapped plasma metabolite ($P< 0.05$) associated with ICB response in B16-F0 tumour-bearing mice and RCC patients with ICB treatment. (**f**) The relative abundance of plasma inosine in tumour-free (n=8) and B16-F0 tumour-bearing mice at day 16 (n=5). (**g**) The relative abundance of plasma inosine in RCC patients with nivolumab treatment at baseline and week 8 (n=160).

Data are presented as Mean ± s.e.m. Statistical significance was determined by Two-sided Student's *t*-test (f, g). The distribution *P*-value was indicated in the figures. Source data are provided as a Source Data file.

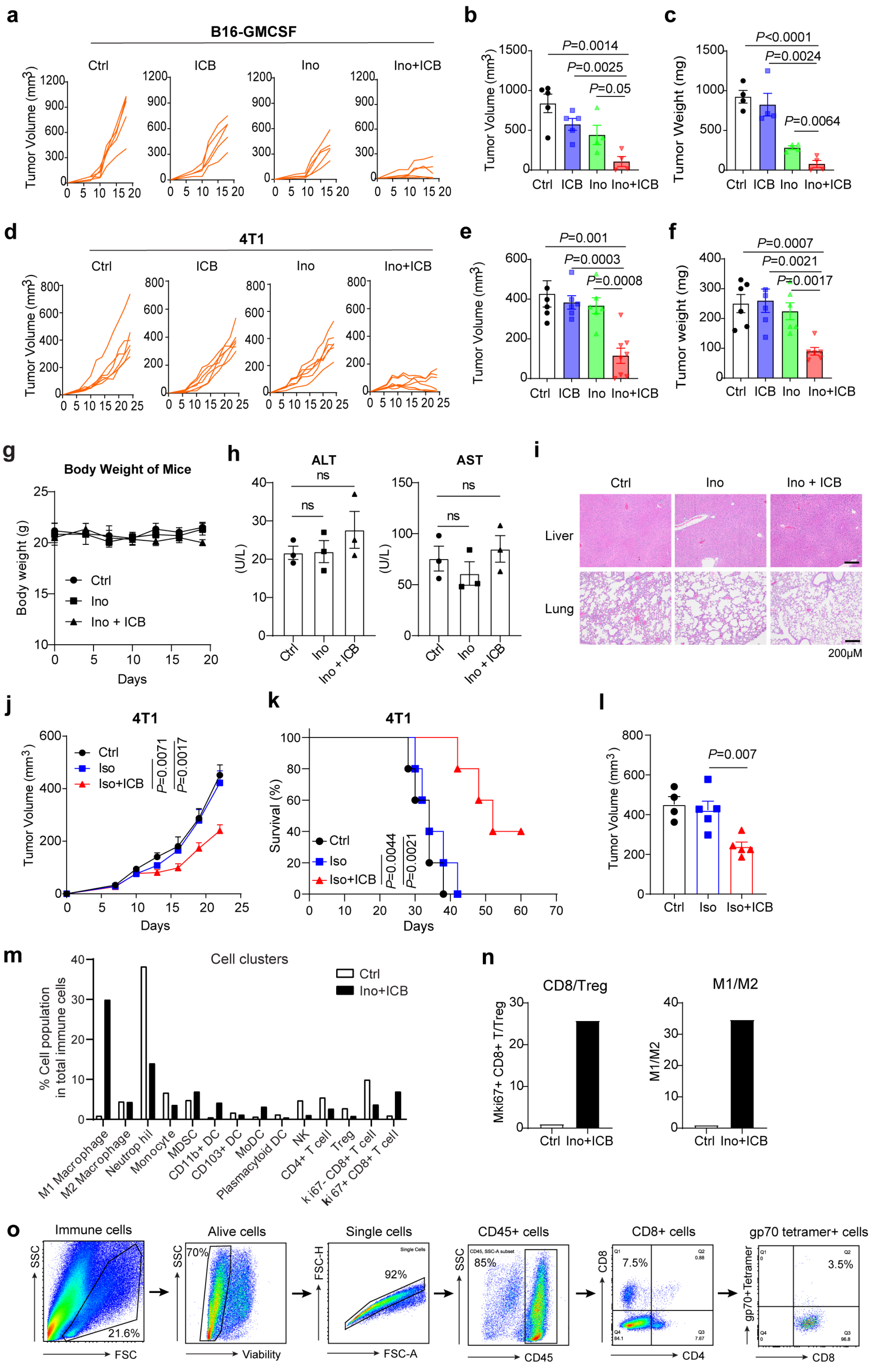
a
B16-GMCSF
Ctrl
ICB
Ino
Ino+ICB
Tumor Volume (mm3)
b
Tumor Volume (mm3)
P=0.0014
P=0.0025
P=0.05
Ctrl ICB Ino Ino+ICB
c
Tumor Weight (mg)
P<0.0001
P=0.0024
P=0.0064
Ctrl ICB Ino Ino+ICB
d
4T1
Ctrl
ICB
Ino
Ino+ICB
Tumor Volume (mm3)
e
Tumor Volume (mm3)
P=0.001
P=0.0003
P=0.0008
Ctrl ICB Ino Ino+ICB
f
Tumor weight (mg)
P=0.0007
P=0.0021
P=0.0017
Ctrl ICB Ino Ino+ICB
g
Body Weight of Mice
Body weight (g)
Ctrl
Ino
Ino + ICB
Days
h
ALT
AST
ns
(U/L)
Ctrl
Ino
Ino + ICB
i
Ctrl
Ino
Ino + ICB
Liver
Lung
200μM
j
4T1
Tumor Volume (mm3)
Ctrl
Iso
Iso+ICB
P=0.0071
P=0.0017
Days
k
4T1
Survival (%)
Ctrl
Iso
Iso+ICB
P=0.0044
P=0.0021
Days
l
Tumor Volume (mm3)
P=0.007
Ctrl Iso Iso+ICB
m
Cell clusters
Ctrl
Ino+ICB
% Cell population in total immune cells
M1 Macrophage
M2 Macrophage
Neutrop hil
Monocyte
MDSC
CD11b+ DC
CD103+ DC
MoDC
Plasmacytoid DC
NK
CD4+ T cell
Treg
k i67- CD8+ T cell
ki 67+ CD8+ T cell
n
CD8/Treg
Mki67+ CD8+ T/Treg
Ctrl Ino+ICB
M1/M2
M1/M2
Ctrl Ino+ICB
o
Immune cells
SSC
FSC
21.6%
Alive cells
SSC
70%
Viability
Single cells
FSC-H
92%
FSC-A
CD45+ cells
SSC
85%
CD45
CD8+ cells
CD8
7.5%
CD4
gp70 tetramer+ cells
gp70+Tetramer
3.5%
CD8

Supplementary Figure 3. Inosine and its derivative Isoprinosine administration strongly enhance the responses to ICB *in vivo*. (**a**) Individual tumour volume of B16-GMCSF tumour-bearing mice with Ctrl, Ino, ICB, or Ino+ICB treatment (n=5). (**b**) Quantification of tumour volume of B16-GMCSF implants as shown in (a) on day 22 (n=5). (**c**) Quantification of tumour weight of B16-GMCSF implants as shown in (a) on day 22 (n=5). (**d**) Individual tumour volume of 4T1 MFP implants in mice with Ctrl, Ino, ICB, or Ino+ICB treatment (n=6). (**e**) Quantification of tumour volume of 4T1 implants as shown in (d) on day 22 (n=6). (**f**) Quantification of tumour weight of 4T1 implants as shown in (d) on day 22 (n=6). (**g**) Changes in body weight over time for all groups from 4T1 tumour-bearing mice with Ctrl, Ino, and Ino+ICB treatment (n=3). (**h**) Quantification of serum alanine aminotransferase (ALT) and aspartate aminotransferase (AST) levels at day 25 from 4T1 tumour-bearing mice with Ctrl, Ino, and Ino+ICB treatment (n=3/group). (**i**) Representative H&E sections of livers and lungs from 4T1 tumour-bearing mice with Ctrl, Ino, and Ino+ICB treatment at day 25. Data were representative of three independent experiments (n=3). (**j**) Tumour volume of 4T1 tumour-bearing mice with IgG2a (Ctrl), 600mg/kg of isoprinosine (Iso), or 600mg/kg of isoprinosine in combination with ICB (Iso+ICB) treatment (n=5). (**k**) Survival of 4T1 tumour-bearing mice with Ctrl, Iso, or Iso+ICB treatment (n=5). (**l**) Quantification of tumour volume of 4T1 tumours as shown in (i) on day 22 (n=5). (**m**) The frequency of 14 identified major immune populations from 4T1 tumour-bearing mice treated with Ctrl (n=16, 199 cells) or Ino+ICB (n=9, 842 cells) treatment. (**n**) The ratio of $CD8^+$ T cells/Treg cells (left) and M1/M2 macrophages (right) in Ctrl and Ino+ICB treated 4T1 tumours. (**o**) Representative flow plots showing the step-by-step gating strategy of $CD8^+$ gp70-$Tetramer^+$ T lymphocytes isolated from 4T1 tumour tissues.

Data are presented as Mean ± s.e.m. Statistical significance was determined by one-way ANOVA and Tukey test for multiple comparisons (b, c, e, f, h, j, l), or log-rank (Mantel-Cox) test (k). The distribution *P*-value is indicated in the figures. Source data are provided as a Source Data file.

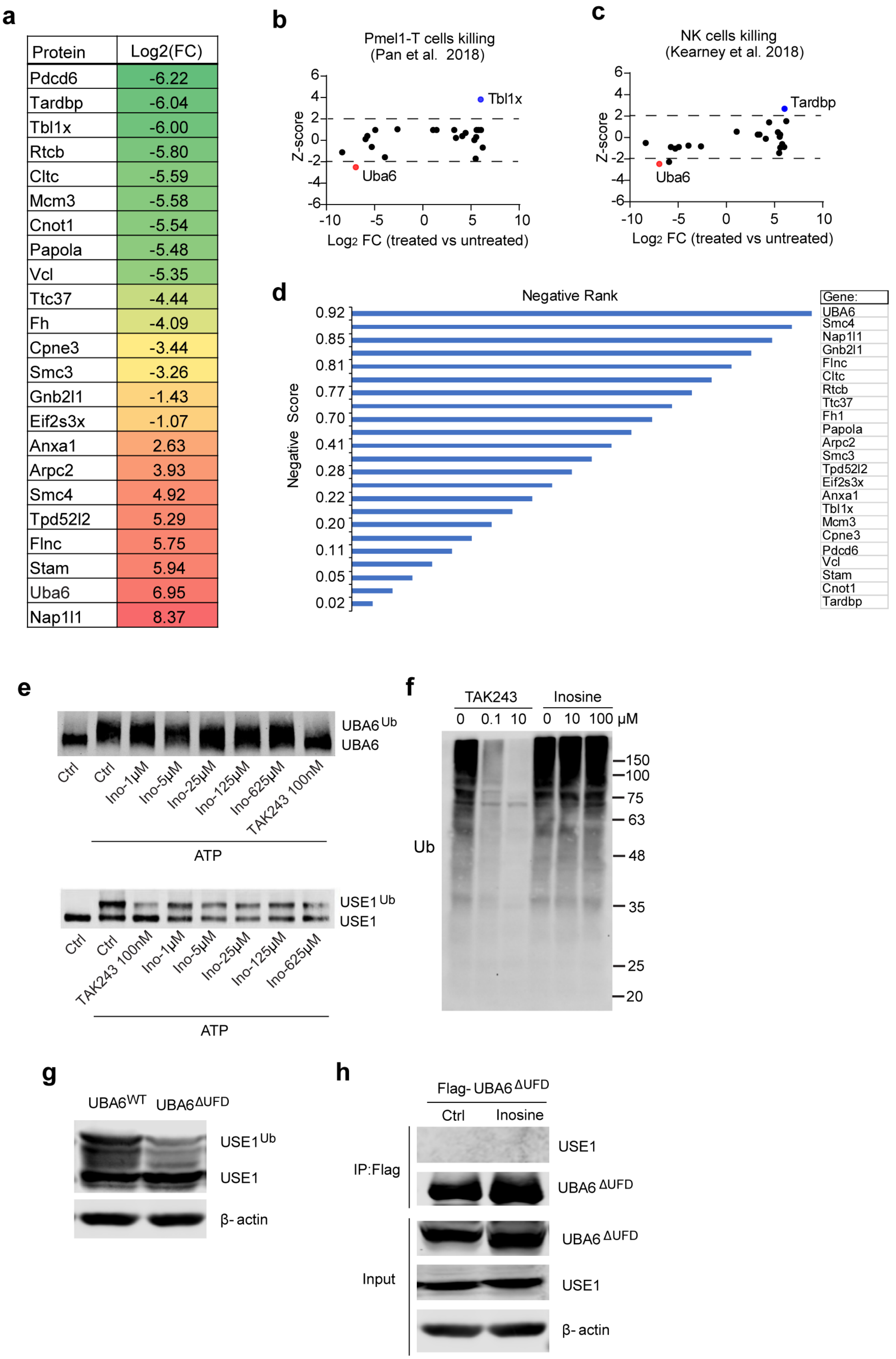

Protein	Log2(FC)
Pdcd6	-6.22
Tardbp	-6.04
Tbl1x	-6.00
Rtcb	-5.80
Cltc	-5.59
Mcm3	-5.58
Cnot1	-5.54
Papola	-5.48
Vcl	-5.35
Ttc37	-4.44
Fh	-4.09
Cpne3	-3.44
Smc3	-3.26
Gnb2l1	-1.43
Eif2s3x	-1.07
Anxa1	2.63
Arpc2	3.93
Smc4	4.92
Tpd52l2	5.29
Flnc	5.75
Stam	5.94
Uba6	6.95
Nap1l1	8.37

Supplementary Figure 4. UBA6 is identified as a target of inosine. (**a**) LiP-SMap screening 23 proteins as inosine-modified candidates. (**b**) The effect of deletion of the top 23 genes in (a) on Pmel1^{+} T-cell mediated tumour killing. A dashed line indicates a z-score of -2 or 2. (**c**) The effect of deletion of the top 23 genes (a) on NK cell-mediated tumour killing. A dashed line indicates a z-score of -2 or 2. (**d**) The bar plot of the analyses of CRISPR screening results based on the screening score in immunocompetent (WT) mice versus immunodeficient (NSG) mice. The higher negative score indicated the high antitumour immune response after gene depletion in tumour cells. (**e**) The effect of inosine on ubiquitination of UBA6 and USE1 protein *in vitro*. Data were representative of two independent experiments. Data were representative of two independent experiments (n=2). (**f**) The effect of inosine on ubiquitination of total proteins in 4T1 cells. TAK243, a UBA1 inhibitor, was used as a positive control. Data were representative of two independent experiments (n=2). (**g**) The deletion of the UFD domain in UBA6 (UBA6$^{\Delta UFD}$) led to the loss of function on USE1 ubiquitination in HEK293 cells. Data were representative of two independent experiments (n=2). (**h**) Co-IP and Western blots showed that UBA6$^{\Delta UFD}$ abolished the impact of inosine on the interaction of UBA6 and USE1in HEK293 cells. Data were representative of two independent experiments (n=2). Source data are provided as a Source Data file.

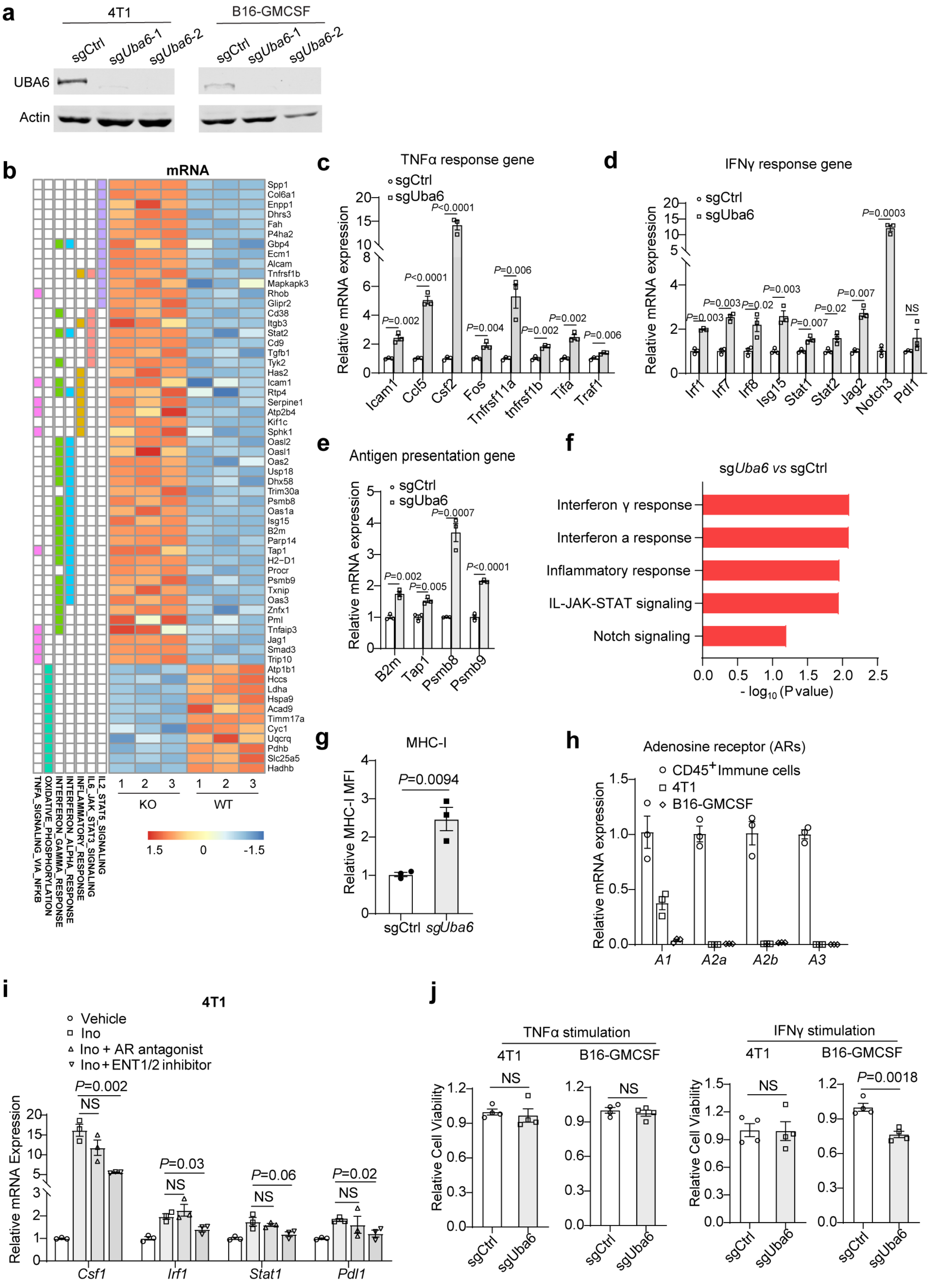

a
4T1
B16-GMCSF
sgCtrl
sgUba6-1
sgUba6-2
UBA6
Actin
b
mRNA
KO
WT
TNFA_SIGNALING_VIA_NFKB
OXIDATIVE_PHOSPHORYLATION
INTERFERON_GAMMA_RESPONSE
INTERFERON_ALPHA_RESPONSE
INFLAMMATORY_RESPONSE
IL6_JAK_STAT3_SIGNALING
IL2_STAT5_SIGNALING
c
TNFα response gene
sgCtrl
sgUba6
Relative mRNA expression
d
IFNγ response gene
e
Antigen presentation gene
f
sgUba6 vs sgCtrl
Interferon γ response
Interferon a response
Inflammatory response
IL-JAK-STAT signaling
Notch signaling
- log10 (P value)
g
MHC-I
Relative MHC-I MFI
P=0.0094
h
Adenosine receptor (ARs)
CD45+ Immune cells
4T1
B16-GMCSF
i
4T1
Vehicle
Ino
Ino + AR antagonist
Ino + ENT1/2 inhibitor
Relative mRNA Expression
j
TNFα stimulation
IFNγ stimulation
Relative Cell Viability

Supplementary Figure 5. UBA6 loss increases inflammatory signaling in tumour cells. (**a**) Expression of UBA6 protein in control (sgCtrl) and *Uba6*-null (sg*Uba6*) 4T1 and B16-GMCSF tumour cells. Data were representative of two independent experiments (n=2). (**b**) Heat-map of gene expression (Red, upregulated; Blue, downregulated) for differential signaling pathway in sgCtrl and sg*Uba6* 4T1 tumour cells. The expression level of these genes in sgCtrl and sg*Uba6* 4T1 cells is measured by RNAseq (n=3). (**c**) The relative mRNA expression of TNFα-response genes in sgCtrl and sg*Uba6* 4T1 tumour cells (n=3). (**d**) The relative mRNA expression of IFNγ-response genes in sg*Uba6* and sgCtrl 4T1 tumour cells (n=3). (**e**) The relative mRNA expression of antigen presentation-related genes in sg*Uba6* and sgCtrl 4T1 tumour cells (n=3). (**f**) Top-ranked GO terms in the proteomics of sg*Uba6* 4T1 tumour cells. (**g**) Flow analyzes the intensity of cell surface MHC-I expression in sgCtrl and sg*Uba6* B16-GMCSF tumour cells treated with IFNγ (10ng/ml) for 48h (n=3). (**h**) The mRNA level of adenosine receptors in 4T1, B16-GMCSF, and $CD45^+$ immune cells isolated from tumour of 4T1 bearing mice. $CD45^+$ immune cells as a positive control. (**i**) The represented antigen processing/presentation and interferon-responsive gene expression in 4T1 tumour cells pretreated with AR antagonist (CGS15943, 0.5μM) or ENT1/ENT2 inhibitor (Dilazep dihydrochloride, 2.0μM) for 24h and then treated with inosine at 100μM for 48h (n=3). (**j**) Cell viability of sgCtrl and sg*Uba6* 4T1 or B16-GMCSF tumour cells following stimulation with 10ng/ml of TNFα or 10ng/ml of IFNγ (n=4).

Data are presented as Mean ± s.e.m. Statistical significance was determined by two-sided Student's *t*-test (c, d, e, j) or one-way ANOVA and Tukey test for multiple comparisons (i). The *P*-values in f were based on log-rank tests. NS, no significant. The distribution *P*-value is indicated in the figures. Source data are provided as a Source Data file.

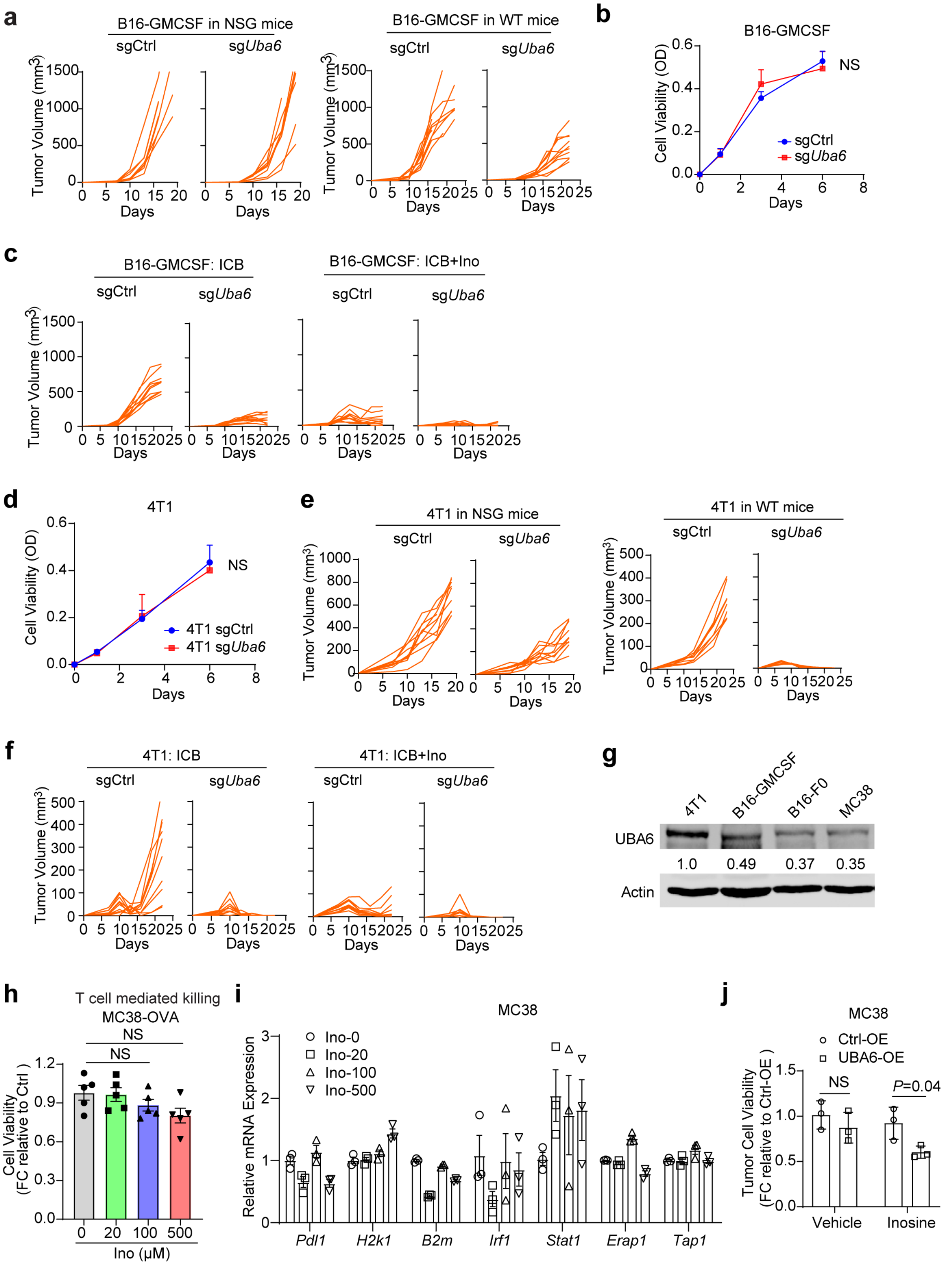
a
B16-GMCSF in NSG mice
sgCtrl
sgUba6
B16-GMCSF in WT mice
Tumor Volume (mm3)
Days
b
B16-GMCSF
Cell Viability (OD)
NS
sgCtrl
sgUba6
Days
c
B16-GMCSF: ICB
B16-GMCSF: ICB+Ino
d
4T1
4T1 sgCtrl
4T1 sgUba6
e
4T1 in NSG mice
4T1 in WT mice
f
4T1: ICB
4T1: ICB+Ino
g
4T1
B16-GMCSF
B16-F0
MC38
UBA6
1.0
0.49
0.37
0.35
Actin
h
T cell mediated killing
MC38-OVA
Cell Viability (FC relative to Ctrl)
Ino (μM)
i
MC38
Relative mRNA Expression
Ino-0
Ino-20
Ino-100
Ino-500
Pdl1
H2k1
B2m
Irf1
Stat1
Erap1
Tap1
j
Ctrl-OE
UBA6-OE
Tumor Cell Viability (FC relative to Ctrl-OE)
P=0.04
Vehicle
Inosine

Supplementary Figure 6. UBA6 loss triggers antitumour immunity and substitutes the effect of inosine in combination with ICB. (**a**) Individual tumour volume of sgCtrl and sg*Uba6*B16-GMCSF tumours in NSG mice (n=10) (left) and WT mice (n=10) (right). (**b**) Cell viability of sgCtrl and sg*Uba6* B16-GMCSF cells by MTT assay (n=3). (**c**) Individual tumour volume of sgCtrl and sg*Uba6* B16-GMCSF tumours in WT mice with ICB (anti-PD-1) (left) or ICB (anti-PD-1) + Ino (right) treatment (n=10). (**d**) Cell viability of control (sgCtrl) and *Uba6*-null (sg*Uba6*) 4T1 cells by MTT assay (n=3). (**e**) Individual tumour volume of sgCtrl and sg*Uba6* 4T1tumours in NSG mice (n=10) (left) and WT mice (n=10) (right). (**f**) Individual tumour volume of sgCtrl and sg*Uba6* 4T1 tumours in WT mice with ICB (left) or ICB + Ino(right) treatment (n=10). (**g**) The protein level of Uba6 in various cancer cell lines was determined by western blot and the quantification of UAB6 protein was shown. Data were representative of two independent experiments (n=2). (**h**) The relative cell viability of MC38-OVA cells was shown. OT-1 T cells were pretreated with indicated concentrations of inosine or vehicle for 24h, then co-cultured with MC38-OVA tumour cells at a 2:1 E: T ratio for 48h (n=5). (**i**) The represented antigen processing/presentation and interferon-responsive gene expression in MC38 tumour cells treated with inosine at indicated concentrations (n=3). (**j**) The relative cell viability of Uba6-overexpressed or control MC38 cells with inosine (100uM) pretreatment for 24h after cocultured with activated T cells for 48h (n=3).

Data are presented as Mean ± s.e.m. Statistical significance was determined by Two-sided Student's *t*-test, and log-rank (Mantel-Cox) test. NS, no significant. The distribution *P*-value was indicated in the figures. Source data are provided as a Source Data file.

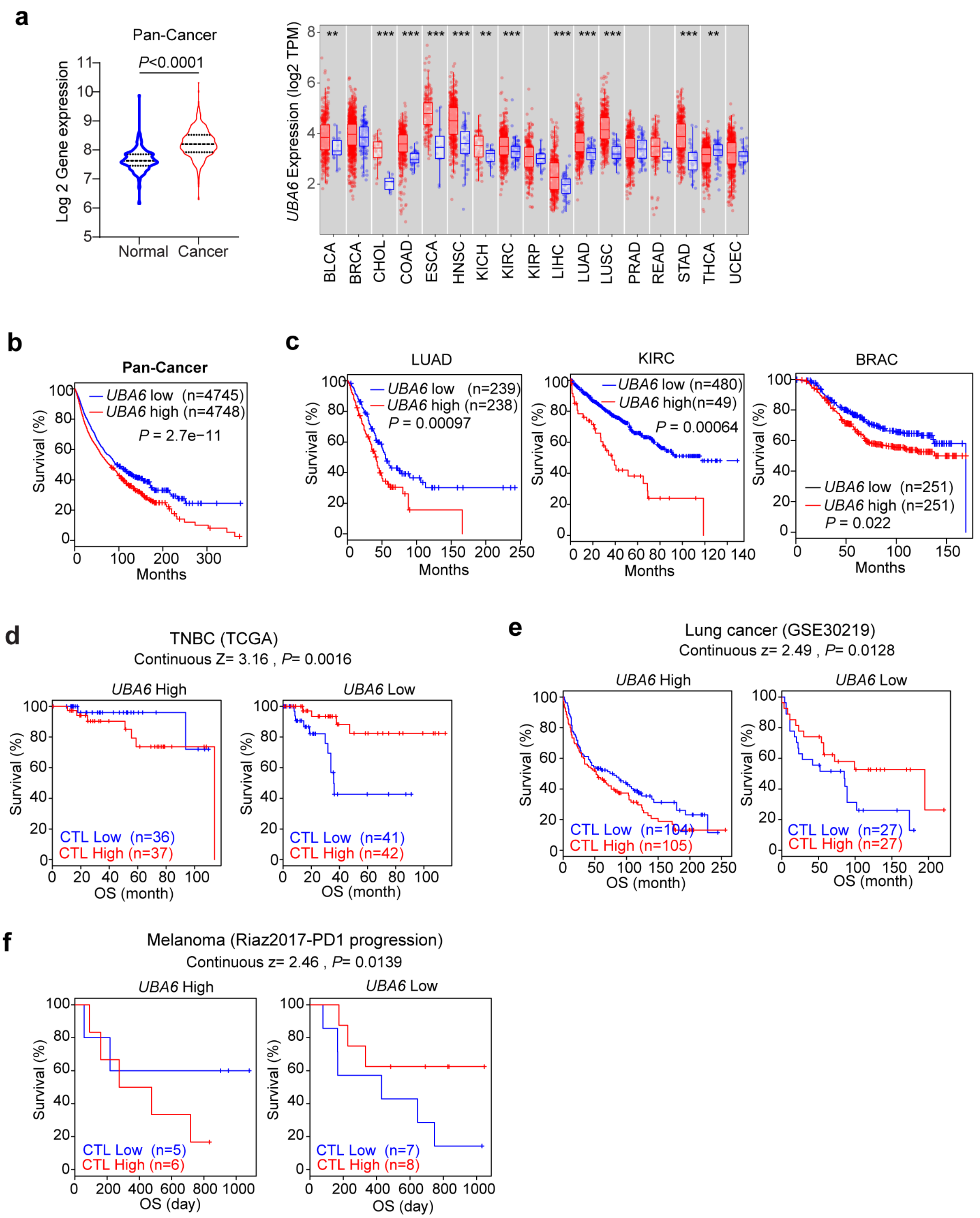
a
Pan-Cancer
P<0.0001
Log 2 Gene expression
Normal
Cancer
UBA6 Expression (log2 TPM)
BLCA
BRCA
CHOL
COAD
ESCA
HNSC
KICH
KIRC
KIRP
LIHC
LUAD
LUSC
PRAD
READ
STAD
THCA
UCEC
b
Pan-Cancer
UBA6 low (n=4745)
UBA6 high (n=4748)
P = 2.7e−11
Survival (%)
Months
c
LUAD
UBA6 low (n=239)
UBA6 high (n=238)
P = 0.00097
KIRC
UBA6 low (n=480)
UBA6 high(n=49)
P = 0.00064
BRAC
UBA6 low (n=251)
UBA6 high (n=251)
P = 0.022
d
TNBC (TCGA)
Continuous Z= 3.16 , P= 0.0016
UBA6 High
CTL Low (n=36)
CTL High (n=37)
UBA6 Low
CTL Low (n=41)
CTL High (n=42)
OS (month)
e
Lung cancer (GSE30219)
Continuous z= 2.49 , P= 0.0128
UBA6 High
CTL Low (n=104)
CTL High (n=105)
UBA6 Low
CTL Low (n=27)
CTL High (n=27)
OS (month)
f
Melanoma (Riaz2017-PD1 progression)
Continuous z= 2.46 , P= 0.0139
UBA6 High
CTL Low (n=5)
CTL High (n=6)
UBA6 Low
CTL Low (n=7)
CTL High (n=8)
OS (day)

Supplementary Figure 7. UBA6 expression is related to survival and cytotoxic T cells activity in cancer patients. (**a**) Violet plot showing the distribution of *UBA6* gene expression in pan-cancer and normal tissues from TCGA. Tissue wide of *UBA6* gene expression patterns across 18 tumours (red box) and paired normal tissues (blue box). Box plots represent the range, median, 25th, and 75th percentile. (**b**) Kaplan-Meier plot analysis of overall survival by the median cut-off of *UBA6* gene expression in pan-cancers (N=9493). (**c**) Kaplan-Meier plot analysis of overall survival by the median cut-off of *UBA6* gene expression in LUAD, KIRC, and BRAC tumours. **(d-f)** The association between the cytotoxic T lymphocytes **(**CTL) level and overall survival for triple-negative breast cancer (TNBC) patients (d), lung cancer patients (e), or anti-PD1 unresponsive melanoma patients (f) with high *UBA6* (left) and low *UBA6* (right) in TCGA. The CTL infiltration level was estimated as the average expression level of *CD8A, CD8B, GZMA, GZMB,* and *PRF1*.

Statistical significance was determined by a Two-sided Student's *t*-test (a) or log-rank (Mantel-Cox) test (b, c, d, e, f). The public data in a-f are available in the TCGA database [https://gdc.cancer.gov/].

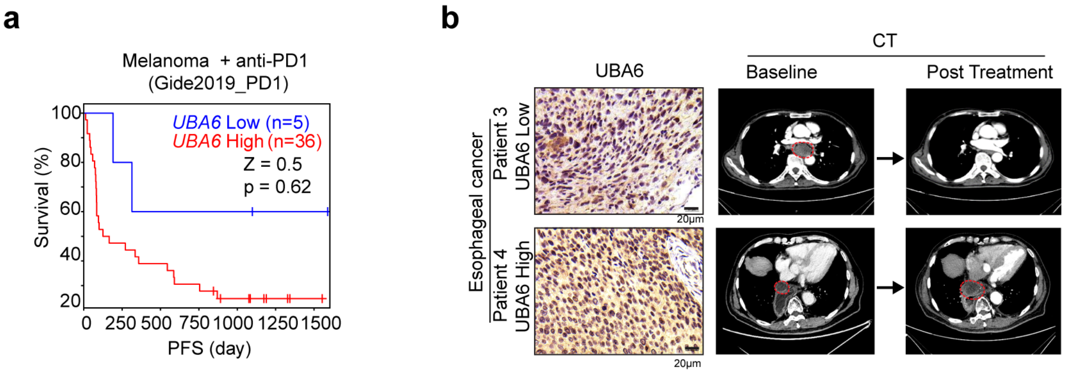

c

UBA6 expression
vs
Response to Anti-PD1

UBA6 in Tumor	Responder (CR/PR/SD)	Non-responder (PD)	Total
Low	8	8	16
High	1	5	6

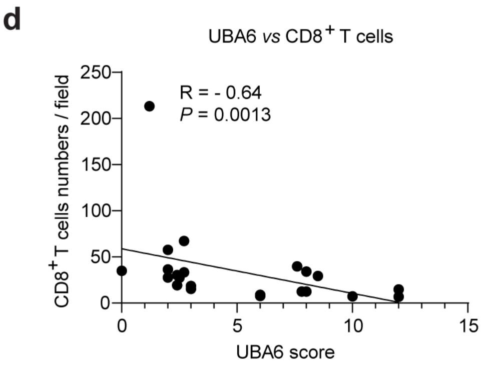

Supplementary Figure 8. UBA6 expression is negatively correlated with immunotherapy responses in cancer patients. (**a**) Kaplan-Meier plots of progress-free survival (PFS) for validated cohorts with 41 melanoma patients treated with anti-PD1 with different *UBA6* levels. Statistical significance was determined by log-rank (Mantel-Cox) test. (**b**) Representative tumour sections immunohistochemically stained for UBA6 protein (left panel, 400x) as well as representative pre-treatment (baseline) and posttreatment (week 8) computed tomographic (CT) scan (right panel) of esophageal cancer Patient #3 (top) and Patient #4 (bottom) treated with an anti-PD1 antibody. Tumour lesions are outlined by a red outline in CT scans. CT scanning of patient#3 (UBA6 low) shows tumour regression after treatment (Baseline: 3.9cm×2.8cm; Posttreatment: 0cm). Patient 4 (UBA6 high) shows tumour progression meaning non-respond to Anti-PD1 based treatment (Baseline: 2.9cm×2.8cm; Posttreatment: 4.8cm×3.8cm). Data were representative of three independent experiments (n=3). (**c**) Distribution of responders or nonresponses' patients with different expression levels of UBA6 in tumour cells to Anti-PD1 treatment anti-PD1 treatment (CR: complete response; PR: partial response; SD: stable disease; PD: progressive disease). (**d**) The correlation of tumoural UBA6 expression score and $CD8^+$ T cells infiltration in the tumour microenvironment. Statistical significance was determined by a nonparametric Spearman correlation test. Source data are provided as a Source Data file.

Population	Key differentially expressed transcripts
M1 Macrophage	*Itgam, Adgre1, C1qa, Cxcl9, Vcam1*
M2 Macrophage	*Itgam, Adgre1, Mrc1, Clec10a, Folr2, Sepp1, Apoe, Maf*
MDSC	*Itgam, Arg1, Nos2, Pf4, Il1rn*
Monocyte	*Itgam, Ly6c2, Il1b, Cxcl10, Ms4a4c*
Neutrophil	*Itgam, Ly6c2, Hp, Msrb1, Ncf2*
CD11b+ cDC	*Itgam, Itgax, Zbtb46, Cd209a, H2-Ab1, H2-Eb1*
MoDc	*Itgam, Clec4b1, Ccl24, Ccr2, H2-Dma, H2-Ab1*
CD103+ cDC	*Flt3, Zbtb46, Batf3, Clec9a, Xcr1, Cd24a, Cd103, H2-Ab1*
Migratory cDC	*Flt3, Zbtb46, Batf3, Ccr7, Relb, Ccl22*
Plasmacytoid DC	*Ccr9, Siglech, Bst2*
Mki67+ CD8+ T cell	*Cd8a, Cd3g, Tox, Cenpe, Cdk4, Thy1*
Mki67- CD8+ T cell	*Cd8a, Cd3g, Tox, Ccl5, Thy1*
NK cell	*Ncr1, Klra7, Klre1*
Treg	*Cd4, Cd3g, Ikzf2, Foxp3*

Supplementary Table 1. Key differentially expressed transcripts that distinguish cell clusters for single-cell RNA-seq experiment.
