# Supplementary material for: Inhibition of UBA6 by inosine augments tumour immunogenicity and responses
Source: Nat Commun. 2022 Sep 15;13:5413. doi: 10.1038/s41467-022-33116-z (PMC9478149; doi:10.1038/s41467-022-33116-z)
Supplement: Supplementary file 3 — Description of Additional Supplementary Files [file 41467_2022_33116_MOESM3_ESM.pdf]

Description of Additional Supplementary Files

File Name: Supplementary Data 1
Description: Identification of 244 plasma metabolites and their relative quantification in B16-bearing mice with IgG2 (Ctrl) or combination of anti-PD1 mAb and anti-CTLA4 mAb (ICB) treatment. Log2 fold change (FC) was calculated as log2 (ICB/Ctrl).

File Name: Supplementary Data 2
Description: Table summarizing the hazard ratios (HR) of High/low each serum metabolite (defined by median) as a predictor in relation to CheckMate 025 RCC patient overall survival using a Cox proportional hazards model.

File Name: Supplementary Data 3
Description: Summary of inosine binding proteins discovered by LiP-SMap approach. Significant changes in the abundance of half-tryptic peptides (fold change > 2 or < 0.5, p < 0.001, > 2 peptides per protein) were as a readout for structural changes induced by binding of inosine.

File Name: Supplementary Data 4
Description: The RNA-seq data for the analysis of control (sgCtrl) and Uba6-null (sgUba6) 4T1 cells.

File Name: Supplementary Data 5
Description: The list of upstream regulators of UBA6 dependent genes in 4T1 tumor cells using IPA.

File Name: Supplementary Data 6
Description: The list of reagents and materials used in this study.
