# Supplementary material for: Inhibition of UBA6 by inosine augments tumour immunogenicity and responses
Source: Nat Commun. 2022 Sep 15;13:5413. doi: 10.1038/s41467-022-33116-z (PMC9478149; doi:10.1038/s41467-022-33116-z)
Supplement: Supplementary file 10 — Reporting Summary [file 41467_2022_33116_MOESM10_ESM.pdf]

nature portfolio

Corresponding author(s): Baokun He

Last updated by author(s): Jul 25, 2022

Reporting Summary

Nature Portfolio wishes to improve the reproducibility of the work that we publish. This form provides structure for consistency and transparency in reporting. For further information on Nature Portfolio policies, see our Editorial Policies and the Editorial Policy Checklist.

Statistics

For all statistical analyses, confirm that the following items are present in the figure legend, table legend, main text, or Methods section.

n/a	Confirmed	
☐	☒	The exact sample size (*n*) for each experimental group/condition, given as a discrete number and unit of measurement
☐	☒	A statement on whether measurements were taken from distinct samples or whether the same sample was measured repeatedly
☐	☒	The statistical test(s) used AND whether they are one- or two-sided<br>*Only common tests should be described solely by name; describe more complex techniques in the Methods section.*
☒	☐	A description of all covariates tested
☐	☒	A description of any assumptions or corrections, such as tests of normality and adjustment for multiple comparisons
☐	☒	A full description of the statistical parameters including central tendency (e.g. means) or other basic estimates (e.g. regression coefficient) AND variation (e.g. standard deviation) or associated estimates of uncertainty (e.g. confidence intervals)
☐	☒	For null hypothesis testing, the test statistic (e.g. *F*, *t*, *r*) with confidence intervals, effect sizes, degrees of freedom and *P* value noted<br>*Give P values as exact values whenever suitable.*
☒	☐	For Bayesian analysis, information on the choice of priors and Markov chain Monte Carlo settings
☒	☐	For hierarchical and complex designs, identification of the appropriate level for tests and full reporting of outcomes
☒	☐	Estimates of effect sizes (e.g. Cohen's *d*, Pearson's *r*), indicating how they were calculated

Our web collection on statistics for biologists contains articles on many of the points above.

Software and code

Policy information about availability of computer code

Data collection	Flow cytometer Canto II was used to run flow samples and data was acquired and analyzed by with FACSDiva version 8. BioTek Synergy 2 Microplate Reader was used for requiring absorbance. Real-time PCR was run on StepOnePlus system (Thermo fisher). RNA-Seq data were de-multiplexed the cellular barcodes and aligned reads to the transcriptome GRCm38. Data was also collected using standard software, such as Microsoft Excel 2016 and GraphPad Prism version 8.
Data analysis	The quantification of peptides and proteins with “label-free quantification” (LFQ) was performed by MaxQuant v1.6.0.1.3. Flow cytometric data analysis was performed using FlowJo version 10. Real-time PCR data was analyzed by StepOne Software v2.2.2. ScRNA-seq data analysis was performed using Cell Ranger Single-Cell Software Suite v2.1.1. Statistical software were run using GraphPad prizm 8.0. Cluster Profiler v 3.18.1 was used to annotate genes with gene ontology (GO) terms and perform GSEA using the Hallmark gene signature collection from mSigDB.

For manuscripts utilizing custom algorithms or software that are central to the research but not yet described in published literature, software must be made available to editors and reviewers. We strongly encourage code deposition in a community repository (e.g. GitHub). See the Nature Portfolio guidelines for submitting code & software for further information.

March 2021

Data

Policy information about availability of data

All manuscripts must include a data availability statement. This statement should provide the following information, where applicable:

- Accession codes, unique identifiers, or web links for publicly available datasets
- A description of any restrictions on data availability
- For clinical datasets or third party data, please ensure that the statement adheres to our policy

The data that support the findings of this study are available from the manuscript and its supplementary information. All source data is provided as supplementary files with this paper. Source data are provided with this paper. Databases used for collecting gene information include the Mouse Genome Informatics database (http://www.informatics.jax.org/) and collecting protein information include the Mouse UniProt FASTA protein database (https://www.uniprot.org/proteomes).

Field-specific reporting

Please select the one below that is the best fit for your research. If you are not sure, read the appropriate sections before making your selection.

☒ Life sciences ☐ Behavioural & social sciences ☐ Ecological, evolutionary & environmental sciences

For a reference copy of the document with all sections, see nature.com/documents/nr-reporting-summary-flat.pdf

Life sciences study design

All studies must disclose on these points even when the disclosure is negative.

Sample size	Sample sizes were determined based on our and other investigators experience with the respective cell lines used (e.g.: Henau et al. 2016 (PMC5634331), Mosely et al. 2017 (PMID: 27923825), Wang et al. 2019 (PMC6558076), Ishizuka et al. 2019 (PMC7241251), Zhong et al. 2020 (PMID: 31898484)). No statistical method was used to predetermine sample size in animal studies.
Data exclusions	Data were not excluded from analysis.
Replication	All the results haven been biologically repeated three times or more. All attempts at replication were successful.
Randomization	Yes, all the animal were randomly to be grouped in all the experiments.
Blinding	The group design and outcome analysis were not performed in a blinded manner, because this is a routine chemistry experiment which is hard to expected.

Reporting for specific materials, systems and methods

We require information from authors about some types of materials, experimental systems and methods used in many studies. Here, indicate whether each material, system or method listed is relevant to your study. If you are not sure if a list item applies to your research, read the appropriate section before selecting a response.

Materials & experimental systems

n/a	Involved in the study	
☐	☒	Antibodies
☐	☒	Eukaryotic cell lines
☒	☐	Palaeontology and archaeology
☐	☒	Animals and other organisms
☐	☒	Human research participants
☒	☐	Clinical data
☒	☐	Dual use research of concern

Methods

n/a	Involved in the study	
☒	☐	ChIP-seq
☐	☒	Flow cytometry
☒	☐	MRI-based neuroimaging

Antibodies

Antibodies used

Anti-UBA6 antibody, Proteintech ,Cat#13211-1-AP, RRID: AB_2211747;
Anti-USE1 antibody, ABclonal, Cat#A7225, RRID: AB_2767774;
Anti-β-Actin antibody, ABclonal, Cat#AC026, RRID:AB_2768234;
The combination of Rat monoclonal anti-CTLA4 antibody (clone 9H10), Bio X Cell, Cat#BE0273, RRID:AB_2687796;
The combination of Rat monoclonal anti-PD1 antibody (clone RPM1-14) , Bio X Cell, Cat#BE0164, RRID: AB_10949609;
Anti-CD16/32 monoclonal antibody (clone 93) , Biolegend ,Cat#101301, RRID:AB_312800;
Anti-CD45-AF 700 (clone 30-F11) , Biolegend, Cat#103127, RRID:AB_493714;
Anti-CD11b-PE (M1/70.15) , Biolegend , Cat#101207, RRID:AB_312790;

	Anti-F4/80-APC (clone BM8), Biolegend, Cat#123115, RRID:AB_893493;<br>Anti-MHC Class II-FITC (clone M5/114.15.2) , Biolegend ,Cat#107605, RRID:AB_313320;<br>Anti-CD206-PE (clone 19.2) , BD Biosciences, Cat#555954, RRID:AB_396250;<br>Anti-CD8-Percp-Cy5.5 (clone 53-6.7) , Biolegend , Cat#100733, RRID:AB_2075239;<br>Anti-CD4-PE (clone RM4-5) antibodies , Biolegend, Cat#100511, RRID:AB_312714;<br>FITC-conjugated anti-mouse H-2Kd antibody (clone SF1-1.1), BioLegend ,Cat#116605, RRID:AB_313740;<br>Anti-CD3 antibody, BioLegend, Cat#100201, RRID:AB_312658;<br>Anti-CD28 antibody , BioLegend, Cat#102101, RRID:AB_312866;<br>Anti-PD1 antibody, Sintilimab, Innovent Biologics, Cat#IBI308;<br>H-2Ld MuLV gp70 Tetramer-SPSYVYHQF-PE, MBL International, Cat # TB-M521-1.
Validation	All antibodies for FACS and western blot were well-recognized clones in the field and validated by the manufacturers. These antibodies are further validated and routinely used in our lab. For FACS staining, all others FMO and isotype controls were used where appropriate and Fc block was added to all immune cells stainings to reduce non-specific staining.

Eukaryotic cell lines

Policy information about cell lines

Cell line source(s)	Human HEK293 cell line<br>Mouse B16-F0, B16-F10, 4T1 and MC38 tumor cell lines
Authentication	Human HEK293 cell line and mouse B16-F0, B16-F10, 4T1 and MC38 tumor cell lines were obtained from the American Type Culture Collection (ATCC). B16-GMCSF cells were generated by retroviral-mediated GM-CSF gene transfer. Cell lines obtained from external institutions were authenticated by morphology, phenotype and growth.
Mycoplasma contamination	All cell lines in our laboratory are routinely tested for mycoplasma contamination and cells used in this study are negative for mycoplasma. Samples were confirmed negative using MycoAlert (Lonza) Mycoplasma Detection Kit.
Commonly misidentified lines<br>(See ICLAC register)	No commonly misidentified cell lines were used in the study.

Animals and other organisms

Policy information about studies involving animals; ARRIVE guidelines recommended for reporting animal research

Laboratory animals	Female WT C57BL/6, BALB/c, and NOD-SCID IL2Rgnull (NSG) mice (6–8 weeks old). All mice were maintained under pathogen-free conditions.
Wild animals	The study did not involve wild animals.
Field-collected samples	The study did not involve samples collected from the field.
Ethics oversight	Animals were maintained according to the ethical and scientific standards of Animal Center at Shanghai General hospital affiliated with Shanghai Jiao Tong University School of Medicine (2019-A012-01).<br>All animal experimental procedures were approved by the Institutional Animal Care and Use Committee of Shanghai General hospital affiliated with Shanghai Jiao Tong University School of Medicine (2019-A012-01).

Note that full information on the approval of the study protocol must also be provided in the manuscript.

Human research participants

Policy information about studies involving human research participants

Population characteristics	A total of 22 human tissue samples were obtained from patients with cancers of esophageal (n=7), stomach (n=4), lung (n=3), colon (n=3), and others (n=5) (gender: 12 males and 10 females; mean ± SD age: 62.4 ± 8.6 years; median age: 63 years; age range, 39–77 years). All 22 cancer patients are recruited for evaluation of anti-PD1 Ab combined with paclitaxel treatment.
Recruitment	None selection had been made, every patient who meet the eligibility criteria were selected for study.
Ethics oversight	This study was conducted according to the Declaration of Helsinki and approved by the Institutional Review Board at Beijing Friendship Hospital affiliated to Capital Medical University (2017-P2-141-02) with written informed consent obtained from all patients.

Note that full information on the approval of the study protocol must also be provided in the manuscript.

Flow Cytometry

Plots

Confirm that:

☒ The axis labels state the marker and fluorochrome used (e.g. CD4-FITC).

☒ The axis scales are clearly visible. Include numbers along axes only for bottom left plot of group (a 'group' is an analysis of identical markers).

☒ All plots are contour plots with outliers or pseudocolor plots.

☒ A numerical value for number of cells or percentage (with statistics) is provided.

Methodology

Sample preparation	Tumors were harvested and minced with scissors before incubation with collagenase A (2 mg/ml, Roche) and DNase I (50 µg/ml, Roche) in RPMI-1640 completed medium (10%FBS, 1% P/S) for 30 min at 37 °C. Tumor samples were homogenized by repeated pipetting and filtered through a 70µm nylon filter (BD Biosciences) in FACS staining buffer (PBS/0.5% albumin) to generate single-cell suspensions. After red blood cell (RBC) lysis (RBC Lysing Buffer, Biolegend), all samples were washed and re-suspended in FACS staining buffer for further flow cytometry analysis.<br>For in vitro analysis of the effect of inosine on MHC Class I antigen expression, B16-GMCSF or 4T1 cells were seeded and treated with a serial dilution of inosine for 48 hours. Cells were non-enzymatically detached from the wells, washed with FACS staining buffer, and then incubated with FITC-conjugated anti-mouse H-2Kd antibody (clone SF1-1.1, BioLegend) for 30 min on ice. After washing, cells were resuspended in FACS staining buffer, and then >2000 cells were analyzed by flow cytometry.<br>For flow cytometry analysis of apoptosis, 4T1 or B16-GMCSF cells were treated inosine for 48h, and following trypsinization and washes in FACS staining buffer, tumor cells were stained for 20 min on ice using the manufacturer's recommended concentrations of Annexin-V PE and 7-AAD from the PE Annexin V Apoptosis Detection Kit 1(BD Pharmingen, USA) according to the manufacturer's instructions.
Instrument	All samples were acquired on Canto II Flow Cytometer (BD Biosciences, USA).
Software	All data were acquired with BD FACSDiva software (BD Biosciences, USA) and all analyses were performed with FlowJo software v10 (BD Biosciences, USA).
Cell population abundance	The frequency of the populations of interest are specified in the appropriate figures (Figure 1g, 2i).
Gating strategy	The cells were gated on FSC-A/SSC-A basis on the location known to contain immune cells and/or tumor cells. Dead cells and doublets were excluded based on forward and side scatters (FSC-A/FSC-H) and Fixable Viability Dye eFluor 506 (Thermo Fisher, USA). To analyze tumor-infiltrated immune cells, live CD45+ followed by population specific markers.

☒ Tick this box to confirm that a figure exemplifying the gating strategy is provided in the Supplementary Information.
